# Two fatal case reports of cardiac tamponade caused by pericardial effusion due to misplaced peripherally inserted central catheters in extremely low birth weight infants

**DOI:** 10.1097/MD.0000000000042822

**Published:** 2025-06-20

**Authors:** Jeongmin Shin, Sae Yun Kim, Young-Ah Youn

**Affiliations:** a Department of Pediatrics, Seoul St. Mary’s Hospital, College of Medicine, The Catholic University of Korea, Seoul, Republic of Korea.

**Keywords:** central venous line, extremely low birth weight infants, pericardial effusion

## Abstract

**Rationale:**

Vascular access is a critical component of care in the neonatal intensive care unit, especially for nutritional support and medication administration. While umbilical vein catheters are commonly used during the first few days of life, peripherally inserted central catheters (PICCs) become the preferred method of central venous access thereafter. However, malpositioning of PICCs, particularly when the tip lies within or in close proximity to the right atrium, may lead to severe complications such as pericardial effusion and cardiac tamponade, especially in extremely low birth weight infants.

**Patient concerns:**

Two extremely low birth weight preterm infants experienced sudden clinical deterioration following PICC insertion, raising concerns about catheter-related complications.

**Diagnoses:**

Both infants were diagnosed with cardiac tamponade secondary to pericardial effusion caused by PICC malposition, with the catheter tip located in or near the right atrium.

**Interventions:**

In both cases, emergency pericardiocentesis was attempted after echocardiographic confirmation of pericardial effusion and clinical signs of tamponade. Despite resuscitative efforts, both infants succumbed to the complication.

**Outcomes:**

The outcome was fatal in both cases and confirmed catheter malposition as the underlying cause of pericardial effusion and cardiac tamponade.

**Lessons:**

To prevent fatal complications such as cardiac tamponade, PICCs should be positioned in safe area. Proper placement in the superior vena cava should be confirmed and maintained. Regular verification of catheter tip position and minimizing unnecessary repositioning are essential for preventing catheter-related adverse events in vulnerable preterm infants.

## 1. Introduction

Vascular access is an essential component of care for critically ill neonates providing nutritional support and administering medications.^[1]^ Umbilical or peripherally inserted central venous catheterization are the frequently performed procedures in neonatal intensive care. While the umbilical vein catheter is usually employed during the initial 3 days, peripherally inserted central catheters (PICCs) are the preferred mode of central venous access once umbilical venous catheters (UVCs) are either discontinued or cannot be inserted. In many cases, PICC insertion is prolonged due to concerns over potential instability, as perceived by clinicians. PICC can be an invasive procedure which can be complicated by various factors. Complications associated with PICC placement include catheter malposition and its associated sequelae, phlebitis, thrombosis, catheter-related bloodstream infections (CRBSIs), catheter occlusion or dysfunction, and unintentional catheter removal. Among these, pleural and pericardial effusions are most commonly attributed to catheter malposition.^[2,3]^ Although the reported incidence varies across studies, malposition and related complications have been observed in 12.56% to 48.2% of cases.^[4,5]^ The incidence of phlebitis has been reported to be approximately 3.6%,^[4]^ while CRBSI rates range from 1.66 to 21.8 episodes per 1000 catheter-days, depending on factors such as the patient population and institutional catheter management protocols.^[6,7]^ Pericardial effusion, though relatively rare with a reported incidence of 0.07% to 2%, may result from catheter malposition and has been associated with a mortality rate of up to 75% in the literature, warranting careful monitoring.^[2,3]^

Pericardial effusion (PCE) is the buildup of fluid between the visceral and parietal layers of the heart which is a relatively rare medical condition in neonates. PCE has various clinical presentations depending on the amount and speed of pericardial fluid accumulation, from asymptomatic to fatal complications, such as cardiac tamponade (CT), which can restrict heart contractility and decrease cardiac output.^[8,9]^ PCE in neonates can be caused by infections (toxoplasma, rubella, cytomegalovirus, herpes or enterovirus), congenital abnormalities, cardiac or pericardial tumor, autoimmune disease, or can be idiopathic or iatrogenic (central venous catheter [CVC]-related complications).^[8–11]^ The most common etiology of PCE in neonates is the presence of an indwelling CVCs, both UVC and PICC.^[1,3]^ Most patients were preterm infants with a gestational age of <30 weeks, extremely low birth weight (<1000 g), and had an intracardiac position or misplaced CVC with an ongoing infusion of parenteral nutrition.^[9]^

Further, PCE/CT after PICC insertion is an life-threatening complication when not promptly diagnosed and expeditiously treated. Therefore, accurately identifying the PICC tip position is crucial to ensure it is correctly placed, allowing the initiation of necessary medications and total parenteral nutrition. A chest X-ray is the most frequently used method to define the position of the PICC tip and is regarded as the gold standard.^[12,13]^ However, X-rays have many disadvantages, such as radiation damage, the time needed for the procedure, and uncertainty in the interpretation of results.^[14]^ Therefore, the use of advanced techniques such as ultrasound-guided localization or intracavitary electrocardiogram (IC-ECG) monitoring should be considered to ensure accurate positioning of the PICC tip.^[15,16]^

The objective of our case report is to present 2 clinical cases of rapidly accumulated PCE and fatal CT in extremely low birth weight infants with displaced PICC. Additionally, we emphasize that adequate and early diagnosis should be done using advanced technique including ultrasonic imaging and IC-ECG.

## 2. Case presentation

### 2.1. Case 1

**Figure 1.**
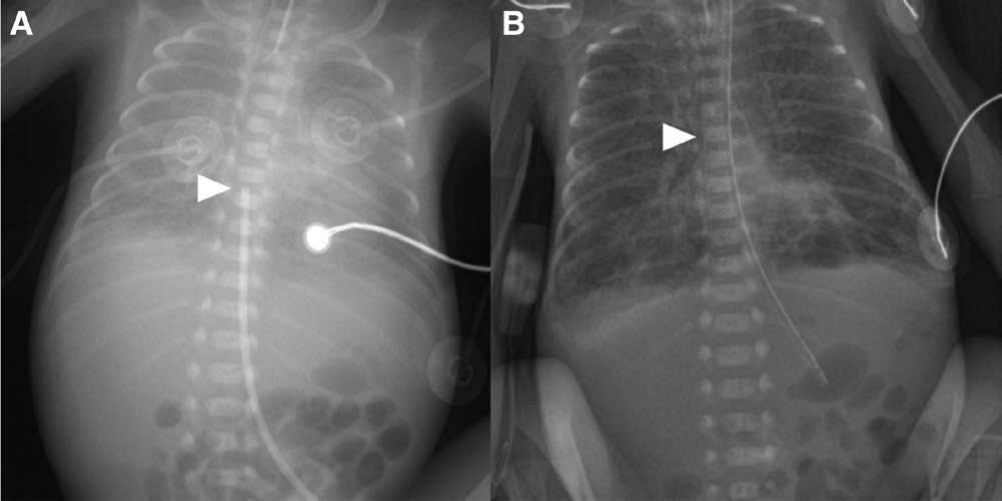
Chest radiography showed the position of initial central lines, case 1 patient. Umbilical venous catheterization was performed immediately after birth. The distal tip UVC was located at T8 level, indicated by a white arrow (A), PICC was inserted at 14th day of life. The position of distal tip of PICC was presumed at cavo-atrial junction (B). PICC = peripherally inserted central catheter, UVC = umbilical venous catheter.

**Figure 2.**
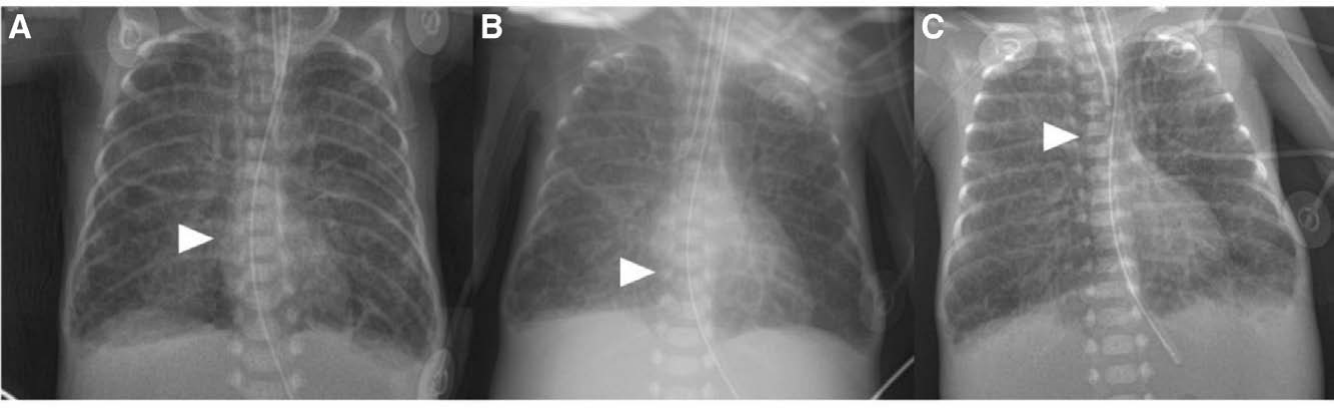
Chest radiographies which were taken before and after cardiopulmonary instability, case 2 patient. Chest radiography was taken on the morning, before the cardiopulmonary deterioration. The distal tip of PICC line lied in border of cardiac silhouette (A), chest radiography immediately after sudden onset of cardiopulmonary deterioration, which showed cardiomegaly with pulmonary congestion compared to the morning chest radiography. The distal tip of PICC line was located in right atrium (B), chest radiography taken after repositioning the PICC, after pericardiocentesis during cardiopulmonary resuscitation. The distal tip of PICC is placed outside of cardiac silhouette. The distal tips of PICC line were indicated by arrow. PICC = peripherally inserted central catheter.

A 592-g (86th centile) male baby was born at 22^2/7^ weeks’ gestation as the first of dichorionic diamniotic (DCDA) twin. A 29 years old, nulliparous woman, his mother was hospitalized due to preterm labor, and emergent cesarean section was performed because of uncontrolled labor. He had Apgar scores of 2 and 6 at 1 and 5 minutes, respectively. Due to the lack of efficient respiratory efforts, he was received a single dose of surfactant and placed on a high-frequency oscillatory ventilator. Immediately after birth, UVC was inserted with appropriate position (Fig. 1A). On day 4, we performed the brain ultrasonography due to reduction of hemoglobin, and intraventricular hemorrhage (IVH), grade 3 was observed, however his respiratory condition was improved till 21 days of life, weaned to conventional ventilation mode. Because of extremely immaturity, we used UVC for prolonged duration without complication. On day 14, a 1 Fr PICC was placed via right median cubital vein, fixed 13 cm from the insertion site. The insertion was uneventful and the tip of PICC was placed at cavo-atrial junction (Fig. 1B), it functioned effectively enabling the administration of parenteral nutrition, and other intravenous medications. Except non-hemodynamic significant patent ductus arteriosus (PDA) and IVH, the hospital stay was uneventful. On day 28, the patient experienced a sudden cardiopulmonary deterioration with bradycardia, cardiopulmonary resuscitation (CPR) was started by the attending neonatologist. Cardiomegaly was identified on chest radiography during resuscitation (Fig. 2) and PCE was confirmed on the bedside echocardiography. An emergent pericardiocentesis was conducted. After drainage of 3.5 mL lipid-like fluid, he recovered from the cardiopulmonary instability. Although return of spontaneous circulation was observed, he could not survive due to multi-organ damage originated cardiac dysfunction and expired the next day.

### 2.2. Case 2

A 793 g (58th centile) female baby was born at 25^5/7^ weeks’ gestation as the second of DCDA twin. This had been an assisted pregnancy with controlled ovarian hyperstimulation, and the mother was 30 years old, nulliparous. Although a cervical cerclage procedure was performed on her mother because of an incompetent internal os of the cervix, preterm labor was not controlled. Therefore, she had to deliver the twin babies after the administration of antenatal corticosteroids.

**Figure 3.**
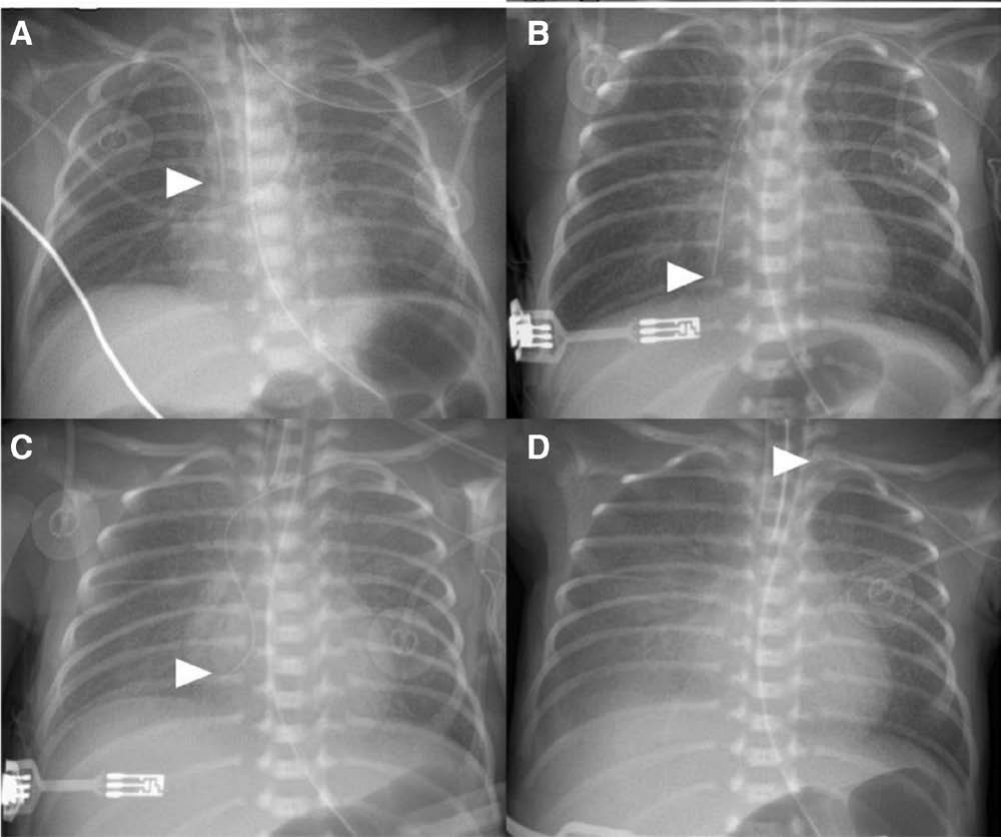
The distal tip of inserted PICC, in case 2 patient. Twelve hours after birth, on the second day of life, the PICC was inserted. The distal tip of the PICC line was identified at the outside of cardiac silhouette (A). The second PICC was inserted on the fiftth day of life. The chest radiography taken immediately after insertion showed the distal tip of PICC line was located inside cardiac silhouette (B), chest X-ray after sudden cardiopulmonary instability showed inappropriate position of the distal tip, curved and deep in the right atrium (C), the PICC was repositioned, the distal tip was placed outside of cardiac silhouette (D). The position of PICC tip was indicated by an arrow. PICC = peripherally inserted central catheter.

The baby needed support with positive pressure ventilation, at the delivery room, and noninvasive ventilation mode was applied, after admission. Due to respiratory difficulty with diffuse granular opacities on chest radiography, she was received a single dose of surfactant for RDS. Twelve hours after birth, a 1 Fr PICC was placed at right median cubital vein, and secured at 14 cm from the insertion site (Fig. 3A). The insertion was uneventful and the tip of PICC was confirmed at the junction of superior vena cava (SVC) and right atrium (RA) on chest radiography. On the third day of life, pulmonary edema and hypotension developed and a hemodynamic significant PDA was diagnosed on echocardiography. After administration of intravenous ibuprofen, the size of PDA decreased and hemodynamic significancy had been improved. On the fifth day of life, initially inserted PICC was removed due to suspicion of leakage. Because the general medical condition was unstable and requiring medication, the attending neonatologists re-inserted a new 1 Fr PICC in into the left axillary vein and fixed at 8 cm from the insertion site, uneventfully. Initially, chest radiography confirmed the distal tip of PICC in the RA, and we repositioned the catheter to cavo-atrial junction, outside the cardiac silhouette. The catheter functioned appropriately (Fig. 3B). Four hours after insertion, she developed sudden hypotension (54 mm Hg and 18 mm Hg, systolic and diastolic blood pressure, respectively), and 3 hours later, heart rate was recorded approximately over 170 bpm. Approximately, 11 hours after insertion, sudden onset of bradycardia was observed from 170 bpm to 60 bpm, without accompanying desaturation. Neonatal CPR was started by the attending neonatologist. After 1 hour of high-quality CPR, the baby did not recover. The tip of PICC was positioned deeply within the RA on chest radiography during CPR (Fig. 3C), and we found out PCE on echocardiography. Urgently, 2 mL of lipid-like pericardial fluid was removed. Then, she recovered to return of spontaneous circulation, after 80 minutes CPR (Fig. 3D). Brain ultrasonography was conducted after this CPR event, this showed grade IV IVH and liquefied intracranial hemorrhage. The remaining hospital stay was uneventful, with the infant being discharged home at 42 weeks of postmenstrual age. However, because of neurologic insult, neurodevelopmental delay was observed. The infant was followed up at pediatrics and rehabilitation medicine outpatient clinics at 10 months of corrected age for prematurity.

## 3. Discussion

The increasing use of PICCs has drawn attention to associated complications, primarily thrombosis and infection among preterm babies. However, many complications related to PICCs further include malposition, and malfunction/occlusion. It is important to place the CVC tip in the correct position to prevent life-threatening complications such as arrhythmias, and erosion of the RA or right ventricle leading to hemothorax, hydrothorax, or CT. For adult patient, the tip of a CVC should be positioned in the proximity of the cavo-atrial junction where the lower third of the SVC and the upper RA is located.^[17,18]^ Several studies addressed safety related to PICC tip location include adult and pediatric populations,^[17,19–21]^ although studies exclusively evaluating neonatal PICCs are rare.^[22]^

Pericardial effusion is one of fatal complication after PICC insertion. In neonatal population, the leading cause of PCE in neonates is CVC-related conditions, including indwelling PICC and UVC. The risk factors associated with PCE are lower gestational age (GA), lower birth weight, intracardiac position or misplaced CVC, and parenteral nutrition infusion.^[1,3,9,23–25]^ In this case report, because the patients were extremely low birth weight with earlier GA, CT occurred with a relatively small amount of pericardial fluid. For reducing these complications, the optimal position for the distal tip of a PICC line in neonates should be in the SVC or inferior vena cave (IVC) but clearly outside the RA, equal to adult patient. The application this rule to clinical cases is not simple, but, Nowlen et al reported the result of study using X-ray that catheter tip position considered appropriate if it remains outside the cardiac silhouette, approximately 2 cm outside the silhouette in term and 1 cm in preterm neonates.^[3]^

Furthermore Warren et al described PCE/CT in neonates with appropriate CVC tip position and they hypothesized that the hyperosmolarity of the parenteral nutrition fluid can damage the endocardium and permeate through the damaged endothelium of the cardiac wall and into the pericardial sac, leading to PCE/CT.^[1]^ Additionally, the repetitive frictional irritation of the catheter tip and chemical irritation of the hyperosmolar fluid damaged the endothelium of the cardiac wall, leading to PCE/CT. These findings support that the distal tip of PICC line should be placed outside the cardiac silhouette. The optimal catheter tip position to avoid complications is the SVC/IVC near the RA, when inserted from the arm or the leg, respectively.^[22,26,27]^ Similarly, the National Health Service Greater Glasgow and Clyde [NHSGGC] neonatology guideline provides a detailed recommendation regarding the optimal positioning of the PICC tip. For a line inserted in an upper limb or scalp vein the tip should be within the SVC but above T4, and for a line inserted in a lower limb the tip should be within the IVC but below T9 and lie to the right side of the spinal column.

Therefore, careful localization of the PICC tip and secure placement using appropriate methods are essential to prevent malposition. Verification of proper catheter tip placement is also important, both at the insertion and during follow-up, as catheter migration can occur. Several cases of PCE have also been reported in patients with properly placed CVCs that may have been caused by catheter migration. Hence, regularly verifying the correct position of the catheter tip is recommended.^[28–30]^ The possibility of displacement of the catheter tip must be excluded, and periodically the tip position should be monitored. Plain radiographs are commonly used to assess PICC tip position; however, they are limited by interobserver variability and a lack of real-time accuracy, particularly in neonates with small anatomical structures. Moreover, variations in patient positioning and projection angle can further compromise the reliability of radiographic interpretation.^[14,31]^ As such, reliance solely on plain radiographs may lead to inaccurate assessment of catheter placement. The position of the upper extremities during chest radiography has been shown to significantly influence the perceived location of the PICC tip. Movements such as arm elevation, adduction, or abduction can cause notable shifts in catheter position, sometimes by several centimeters, potentially leading to misinterpretation of displacement and unnecessary repositioning.^[32,33]^ Inconsistencies in arm positioning at the time of imaging may therefore contribute to variability in radiographic assessments. To enhance the reliability of tip localization, it is advisable to standardize upper limb positioning during radiographic evaluations. Given the limited accuracy of plain radiographs in confirming PICC tip position and the associated radiation exposure, consideration should be given to the use of advanced imaging or guidance modalities to enhance safety and precision. Ultrasonic imaging and IC-ECG may be a useful noninvasive modalities in detecting tip position, performing real-time manipulation, and minimizing radiation exposure.^[8,15,16,34]^

Ultrasound has emerged as a valuable tool for confirming the position of PICC tips in neonates. As a real-time, radiation-free modality, ultrasound allows direct visualization of the catheter tip within the superior vena cava (SVC) or right atrium (RA), facilitating immediate repositioning if necessary. This is particularly advantageous in preterm infants, who are more vulnerable to radiation exposure and have smaller anatomical structures that make precise catheter placement more challenging. Several studies have demonstrated the accuracy and safety of ultrasound in identifying catheter tip location, with diagnostic performance comparable to or exceeding that of conventional radiography.^[15,35]^

Intracavitary electrocardiogram monitoring offers an alternative or complementary method for guiding and confirming PICC tip placement in neonates. The technique relies on characteristic changes in the P-wave amplitude as the catheter approaches the cavo-atrial junction, enabling precise localization without the need for imaging. Recent evidence suggests that ECG-guided placement improves first-attempt success rates and reduces the need for catheter repositioning, thereby lowering the risk of procedure-related complications.^[34,36]^ Given its simplicity, low cost, and repeatability, ECG guidance is increasingly being adopted in neonatal intensive care settings as part of a multimodal approach to optimize catheter placement.^[37]^

## 4. Conclusions

It is not always possible to advance a PICC line to the predetermined position and it may be necessary to use a PICC line which lies more peripheral (within a large vein), or central (within cardiac silhouette), than the optimal position, however the physicians must be aware of the increased risk of malposition under these circumstances. Most importantly, PICCs should be avoided in positioning in the RA, as this can lead to irritation or injury to the cardiac wall in very preterm babies, increasing the risk of CT due to PCE which can be fatal.

In conclusion, for preventing the fatal complication of PICC insertion including CT, accurate placement of the PICC tip in the appropriate anatomical position is essential, and regular monitoring for potential PICC malposition is recommended to ensure continued proper catheter positioning. PICC catheters that are no longer medically indicated should be promptly removed. In addition, the use of advanced techniques for confirming PICC tip position should be considered to enhance placement accuracy and patient safety.

## Author contributions

**Conceptualization:** Young-Ah Youn.

**Data curation:** Jeongmin Shin, Sae Yun Kim.

**Formal analysis:** Jeongmin Shin, Sae Yun Kim.

**Investigation:** Jeongmin Shin, Young-Ah Youn.

**Project administration:** Jeongmin Shin, Young-Ah Youn.

**Resources:** Jeongmin Shin.

**Supervision:** Young-Ah Youn.

**Validation:** Young-Ah Youn.

**Writing – original draft:** Jeongmin Shin, Sae Yun Kim, Young-Ah Youn.

**Writing – review & editing:** Sae Yun Kim, Young-Ah Youn.
